# Peak and averaged bicoherence for different EEG patterns during general anaesthesia

**DOI:** 10.1186/1475-925X-9-76

**Published:** 2010-11-20

**Authors:** Stacey Pritchett, Eugene Zilberg, Zheng Ming Xu, Paul Myles, Ian Brown, David Burton

**Affiliations:** 1 Electrical and Computer Science Engineering, Monash University, Clayton, Vic, Australia; 2 Medical Innovations, Compumedics Pty Ltd, Abbotsford, Vic, Australia; 3 Anaesthesia and Perioperative Medicine, Alfred Hospital, Prahran, Vic, Australia; 4 Medicine, Nursing and Health Sciences (Central Clinical School), Monash University, Clayton, Vic, Australia

## Abstract

**Background:**

Changes in nonlinear neuronal mechanisms of EEG generation in the course of general anaesthesia have been extensively investigated in research literature. A number of EEG signal properties capable of tracking these changes have been reported and employed in anaesthetic depth monitors. The degree of phase coupling between different spectral components is a marker of nonlinear EEG generators and is claimed to be an important aspect of BIS. While bicoherence is the most direct measure of phase coupling, according to published research it is not directly used in the calculation of BIS, and only limited studies of its association with anaesthetic depth and level of consciousness have been published. This paper investigates bicoherence parameters across equal band and unequal band bifrequency regions, during different states of anaesthetic depth relating to routine clinical anaesthesia, as determined by visual inspection of EEG.

**Methods:**

41 subjects scheduled for day surgery under general anaesthesia were recruited into this study. EEG bicoherence was analysed using average and smoothed-peak estimates calculated over different regions on the bifrequency plane. Statistical analysis of associations between anaesthetic depth/state of consciousness and bicoherence estimates included linear regression using generalised linear mixed effects models (GLMs), ROC curves and prediction probability (P_k_).

**Results:**

Bicoherence estimates for the δ_θ region on the bifrequency plane were more sensitive to anaesthetic depth changes compared to other bifrequency regions. Smoothed-peak bicoherence displayed stronger associations than average bicoherence. Excluding burst suppression and large transients, the δ_θ peak bicoherence was significantly associated with level of anaesthetic depth (z = 25.74, p < 0.001 and R^2 ^= 0.191). Estimates of P_k _for this parameter were 0.889(0.867-0.911) and 0.709(0.689-0.729) respectively for conscious states and anaesthetic depth levels (comparable BIS estimates were 0.928(0.905-0.950) and 0.801(0.786-0.816)). Estimates of linear regression and areas under ROC curves supported P_k _findings. Bicoherence for eye movement artifacts were the most distinctive with respect to other EEG patterns (average |z| value 13.233).

**Conclusions:**

This study quantified associations between deepening anaesthesia and increase in bicoherence for different frequency components and bicoherence estimates. Increase in bicoherence was also established for eye movement artifacts. While identified associations extend earlier findings of bicoherence changes with increases in anaesthetic drug concentration, results indicate that the unequal band bifrequency region, δ_θ, provides better predictive capabilities than equal band bifrequency regions.

## Background

Monitoring depth of anaesthesia during surgery is of increasing interest [1,2]. It aims to reduce the risks of intra-operative awareness and post-operative recall, as well as the effects associated with over-anaesthetisation, such as hypotension, delayed recovery, and possibly long term mortality [3].

Spontaneous electroencephalogram (EEG) is a commonly used physiological indicator of the state of consciousness or anaesthetic depth [4]. This is possibly due to the various modulating effects of anaesthetic drugs on the neuronal mechanisms, which in turn are reflected in the EEG. Some of these effects manifest themselves as changes in the power distribution of the EEG frequency components, which enables the application of spectral analysis for determination of anaesthetic depth. However, the complexities of the EEG patterns associated with the drug effects are such that the full range of anaesthetic depth cannot be reliably mapped using a continuum of univariate spectral values [5]. An anaesthetic depth scale based on the appearance of characteristic EEG patterns in the time domain has been proposed [6]. The application of these scales for computerised clinical monitoring is limited by challenges of algorithm implementation and reported presence of nonlinear neuronal mechanisms [7] that are sensitive to the anaesthetic depth and state of consciousness, but cannot be captured in visual interpretation.

The degree of phase coupling between different spectral components is one marker of such nonlinear EEG generators, and can be estimated using the bispectral analysis. The BIS monitor (Aspect Medical), uses a combination of burst-suppression detection, spectral and bispectral analyses to deliver a scalable value that relates to depth of anaesthesia: the bispectral index [8]. However, the bispectral analysis parameter used by BIS is the bispectrum, which by definition reflects not only the degree of phase coupling, but also the EEG amplitude. The bicoherence represents the normalization of the bispectrum, and as such is independent of signal amplitude, therefore providing a more appropriate measure of phase coupling. For further description of bicoherence [see Additional file 1].

Bicoherence is not the only non-linear EEG processing technique applied for determination of consciousness states and anaesthesia levels. Approximate entropy [9], spectral entropy [10] and recently introduced permutation entropy [11,12] were found to be associated with changes in anaesthetic depth, thus further establishing the role of non-linear EEG generation mechanisms in anaesthesia.

A thorough explanation of how the bicoherence changes across the whole bifrequency plane with anaesthetic depth, and how these changes actually relate to the patterns seen in the EEG, is currently unavailable. Earlier studies applying bispectral analysis to the monitoring of depth of anaesthesia [13-18] have shown an increase in the bicoherence values in the lower frequency regions (below 5Hz) at pre-incision (sedated) compared to pre-induction (awake) EEG. More recently Hagahira et al [19-21], Morimoto et al [22] and Hayashi et al [23,24], have completed work describing the influences anaesthesia has on bicoherence of the spontaneous EEG. This work has all used the same approach, and has investigated the changes related to anaesthetic concentration along the diagonal (f1 = f2) of the bicoherence plot (i.e. in the equal band bifrequency regions) [20-22] and were able to define the occurrence of two peaks (referred to as peak-bicoherence, pBIC). These peaks were pBIC-low (between 2 and 6 Hz) and pBIC-high (between 7 and 13 Hz). They also discovered a third peak, which was noted to occur along the ridge of α frequencies, i.e. the set of bicoherence values that occur at BIC(α, all other frequencies), which always occurred at BIC(α, 2α), indicating the non-linear phase coupling between a base frequency (α) and its 1st (2α) and 2nd (3α) harmonics (i.e. the unequal band bifrequency regions). It has been noted that there is also a broad peak occurring along the ridge relating to the pBIC-low peak, however the changes relating to this ridge were not thoroughly investigated. These findings highlight the importance of including the entire bifrequency plane during bispectral analysis, as while studying the diagonal will provide insight into the first harmonic that may be produced by the generation of one frequency, it neglects any higher harmonics that may be present, or any nonlinear coupling that may occur between patterns representing two different frequencies.

To achieve balanced anaesthesia in the clinical environment, it is common practice to use a combination of different hypnotic and analgesic agents [25]. So far, analysis of the bicoherence in depth of anaesthesia monitoring has focused on changes relating to the concentration of one individual anaesthetic regimen at a time. Although each hypnotic or analgesic agent will have known effects on the EEG with deepening anaesthesia, these effects occur at different concentrations between different agents [4]. Minimum alveolar concentration (MAC) values have been determined for each inhalational anaesthetic agent so that agents may be compared directly in relation to desired clinical endpoints. However, MAC represents the value at which 50% of subjects respond to skin incision, therefore largely reflecting effects at the spinal level. Due to this choice of clinical end-point, it has been suggested that the hypnotic effect on the EEG may not be synchronous with the analgesic and immobility effects reflected by the MAC measurement, resulting in different effects on the EEG at equi-MAC levels of different hypnotic agents [26]. It is also known that inter-subject differences in pharmacokinetics and pharmacodynamics may cause variability in EEG responses at equivalent anaesthetic dosage [27], and that external factors during clinical anaesthesia, such as noxious stimulus, can cause varying levels of arousal in the EEG at constant anaesthetic dosage [9].

Therefore in order to build upon the fundamental agent and dose-specific research that has already been undertaken [19-24], and to bridge the knowledge gap of how these findings relate to routine general anaesthesia, it is of interest to understand how the different bicoherence related parameters will change with the different known patterns encountered in the EEG during general anaesthesia, and whether it is more beneficial to apply bicoherence analysis across equal band (i.e. δ_δ) or unequal band (i.e. δ_θ) bifrequency regions with respect to tracking depth of anaesthesia.

The premise of this study was that important information from the bicoherence plot is lost when the analysis focuses only on the equal band bifrequency regions with respect to depth of anaesthesia monitoring in the setting of routine clinical anaesthesia. Bicoherence was investigated using different visually determined EEG patterns relating to anaesthetic depth. It was expected that the bicoherence changes would be consistent with those seen in association with variation in anaesthetic concentration. This study has expanded on previous research by including the whole bifrequency plane (both equal band and unequal band bifrequency regions) and by comparing two different analysis estimates, the averaged and the smoothed-peak bicoherence values, for different regions on the bifrequency plane. This analysis method also gives the flexibility of investigating some EEG patterns that are traditionally viewed as artifacts or abnormal EEG during routine monitoring, such as muscle activity, eye movements and burst suppression.

## Methods

### Patient selection

After obtaining ethics approval from the Alfred Hospital (Melbourne, Australia), 41 patients were included in the study. Selection criteria included adults aged 18 and above, undergoing day surgery with general anaesthesia, excluding procedures on the head. Patients did not receive pre-medication and anaesthetic practice was at the discretion of the anaesthetist. Surgery duration varied from 30 min to 2 hrs depending on surgery type.

### Data generation

EEG was recorded throughout surgery from prior to induction until after first response on awakening. Data was obtained from the BIS A-2000 unit, with XP platform (Aspect Medical Systems, Natick, MA), using the standard BIS sensor placed on the forehead as per manufacturer's instructions and smoothing set to 15 seconds. Raw EEG was recorded with a 128 Hz sampling rate and filtered to remove mains (50 Hz) electrical artifact.

Data was analysed using non-overlapping, consecutive 1 minute segments of raw EEG. Segment length was chosen to achieve a balance between reductions in bicoherence variability [19] and practicality associated with minimising disturbance to patient surgery. Each 1 minute segment of raw EEG was visually inspected and any segments with excessive artifacts were excluded from further analysis. Excessive artifacts included instances of sharp or abrupt amplitude changes and any period where non-biological influences overloaded the BIS recording amplifier, resulting in clipping or disappearance of physiological EEG (i.e. during diathermy). For the purpose of this study, eye blinks were considered a sign of the conscious state and therefore were not processed as artifacts. The occurrence of movement was determined to be an artifact if it was seen at any point during deep anaesthesia, and only if it was of excessive amplitude during the waking period.

**Figure 1:**
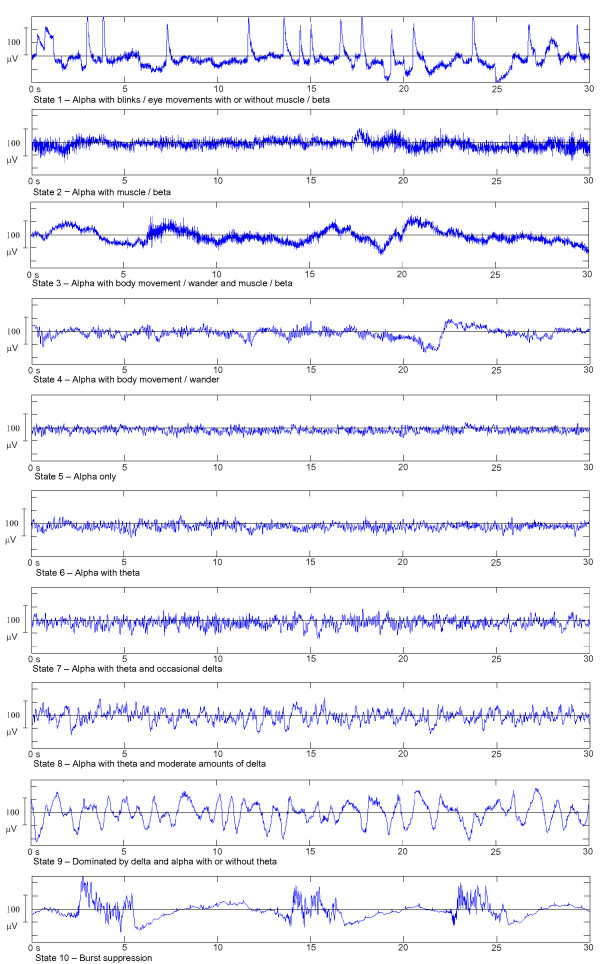
**Examples of EEG states relating to deepening sedation**. Ranging from awake (states 1 - 4) to burst suppression (state 10). The raw EEG is recorded via ASCII protocol from the BIS unit. These are typical examples from the current dataset and therefore from an anaesthetic regimen of bolus induction (either propofol or thiopentone) and inhalational maintenance (sevoflurane, isoflurane or desflurane).

The segments were then visually classified into 10 EEG states relating to the patterns observed within them. These states were selected based on work described by Kugler [28], Schultz et al [29,30] and Bennett et al [31], reflecting the changes seen in the selected patients. States 1 to 4 (Figure 1) show examples of the conscious EEG with varying amounts of movement, EOG, muscle artifact and beta activity present. States 5 - 9 represent deepening levels of anaesthesia and show increasing presence of slower EEG activity. State 10 represents the deepest level of anaesthesia recorded, and shows burst suppression episodes of varying duration.

For comparison of consciousness versus unconsciousness based on visual interpretation of the EEG, states 1-4 and states 5-10 were used respectively. As there were two EEG states (eye movements and burst suppression) whose bicoherence values did not appear to follow the trend of the rest of the states, analysis was performed as either including or excluding eye movements and burst suppression. For investigating graduated changes in anaesthetic depth, states 2-4 were used as a single conscious level, and the remaining states represented deepening levels of anaesthesia. The states of eye movement (state 1) and burst suppression (state 10) were not included when assessing graduated responses.

EEG bicoherence was calculated in MATLAB following the described equations [see Additional file 1]. Values were calculated for each classified 1 minute segment of raw EEG using 2 second epochs with a 75% overlap. Epoch length and overlap were chosen to coincide with those values reportedly used by the BIS monitor [8].

**Figure 2:**
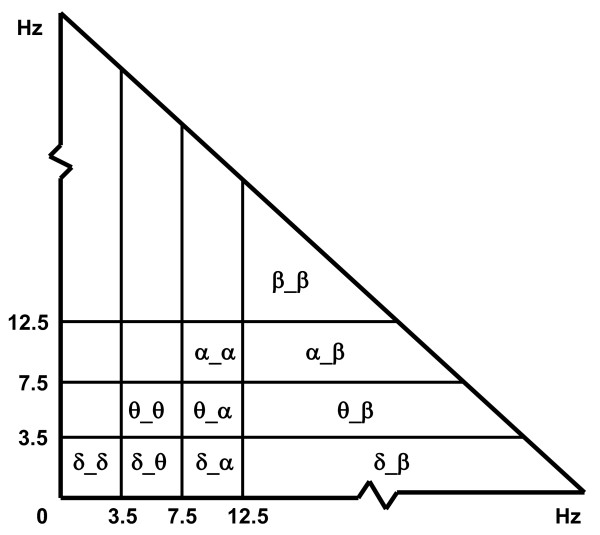
**Bifrequency regions used to calculate average and peak bicoherence values**. As the bicoherence plane is symmetrical about the diagonal, only one side needs to be calculated. Frequency resolution used was 0.5 Hz.

In order to analyse the interactions between frequency bands, the bicoherence was divided into bifrequency regions labelled δ_δ, δ_θ, δ_α, δ_β, θ_θ, θ_α, θ_β, α_α, α_β and β_β, as presented in Figure 2. Both average and smoothed-peak bicoherence values were calculated for each bifrequency region. The average bicoherence was calculated as the average value across a designated bifrequency region. The smoothed-peak bicoherence was estimated as the maximum value across a designated bifrequency region after smoothing. Smoothing was performed in order to reduce spurious data, and was achieved by averaging the bicoherence values for each given point on the bifrequency plane with all its adjoining neighbours. It is useful to compare these two processing methods in order to understand the effects of underlying versus localised bicoherence estimates in a given bifrequency region [32].

BIS index values were automatically recorded every second, and as a basis of comparison with the bicoherence data, an averaged BIS index value was calculated for each classified 1 minute segment of EEG.

### Statistical analyses

Statistical analysis was conducted to address a number of questions relevant to understanding the bicoherence changes during anaesthesia. Firstly, are there significant differences between bicoherence estimates for conscious and unconscious states, and what is the performance of the bicoherence based diagnostic test to discriminate between these states. Secondly, are the bicoherence estimates associated with increase in anaesthetic depth? And finally, what is the behaviour of bicoherence during those states that do not follow the trend of spectral changes with deepening anaesthesia (primarily eye movement artifacts and burst suppression).

The difference in bicoherence estimates of consciousness versus unconsciousness was investigated using linear regression based on univariate generalised mixed effects model (GLM) with random intercepts. In these models an additional random error term (random intercept) was introduced as a constant for all one minute segment observations from the same study to take into account potential correlation between these observations. The accuracy of discriminating between conscious and unconscious states was further assessed through the use of areas under the ROC curve and prediction probabilities (P_k_), with comparison to that of the BIS index values. The P_k _values and corresponding confidence intervals were found using the jackknife method as described by Smith et al [33], and the area under the ROC curve values were compared using the method described by Hanley and McNeil [34] for data derived from the same cases.

Univariate random intercept GLMs were also used to investigate the associations between changes in the level of anaesthesia and bicoherence estimates of each bifrequency region. The ability of using bicoherence to track graduated levels of anaesthesia was investigated by estimating P_k _and comparing these P_k _values to those using the BIS index.

The differences between the bicoherence estimates during eye movement artifacts and burst suppression versus other physiological states, was also examined using respective univariate random intercept GLMs.

P values of < 0.05 were considered statistically significant. Where appropriate, results are expressed as mean (95% confidence interval). Statistical analysis was performed using Intercooled Stata 9 and with the gllamm program written for Stata [35]. Results from gllamm were confirmed using the PROC MIXED function in SAS.

## Results

41 patients (20 men, 21 women) were recruited into this study, with age of 54 ± 19 years. Further patient demographics, including anaesthetic agents, are presented in table 1. The majority of the patients (36/41, or 83%) received similar anaesthetic regimen, with propofol bolus induction and sevoflurane maintenance. The sample sizes for the classified EEG states are summarised in table 2. For examples of the raw EEG for each state, refer to Figure 1.

**Table 1:** Patient Demographics and Anaesthetic Procedure.

Age (yrs)*	54 (19 - 90)
Male sex	20 (49%)
BMI*	29.5 (16.1 - 45.1)
Body temperature (°C)*	36.4 (35 - 38.6)
Duration of surgery (min)*	52 (10 - 154)
**ASA status**	
I	7 (17%)
II	22 (54%)
III	11 (27%)
IV	1 (2%)
**Opioids**	
Fentanyl	35 (85%)
Morphine	6 (15%)
**Induction**	
Midazolam	16 (39%)
Propofol	40 (98%)
Thiopentone	1 (2%)
**Maintenance**	
Sevoflurane	38 (93%)
Isoflurane	2 (5%)
Desflurane	1 (2%)
**Nitrous Oxide**	6 (15%)
**Muscle Relaxants**	26 (63%)

Data are number of patients (%) unless otherwise stated.

*Data are mean (range).

Anaesthetic regimen was at the discretion of the anaesthetist. 36 patients received a similar regimen of propofol induction and sevoflurane maintenance.

**Table 2:** Summary of EEG states with sample sizes.

EEG state	Description	Sample size	
		Number of observations	Number of subjects
1	Alpha with blinks/eye movements ± muscle/beta	13	8
2	Alpha with muscle/beta	16	6
3	Alpha with body movement/wander and muscle/beta	105	36
4	Alpha with body movement/wander	48	11
5	Alpha only	11	5
6	Alpha with theta	14	4
7	Alpha with theta and occasional delta (≤ 25% delta)*	444	31
8	Alpha with theta and moderate delta (25 ≥ 75% delta)*	999	40
9	Dominated by delta and alpha ± theta (> 75% delta)*	159	24
10	Burst suppression	61	14

*This percentage is based on the time occupied by the delta activity.

**Figure 3:**
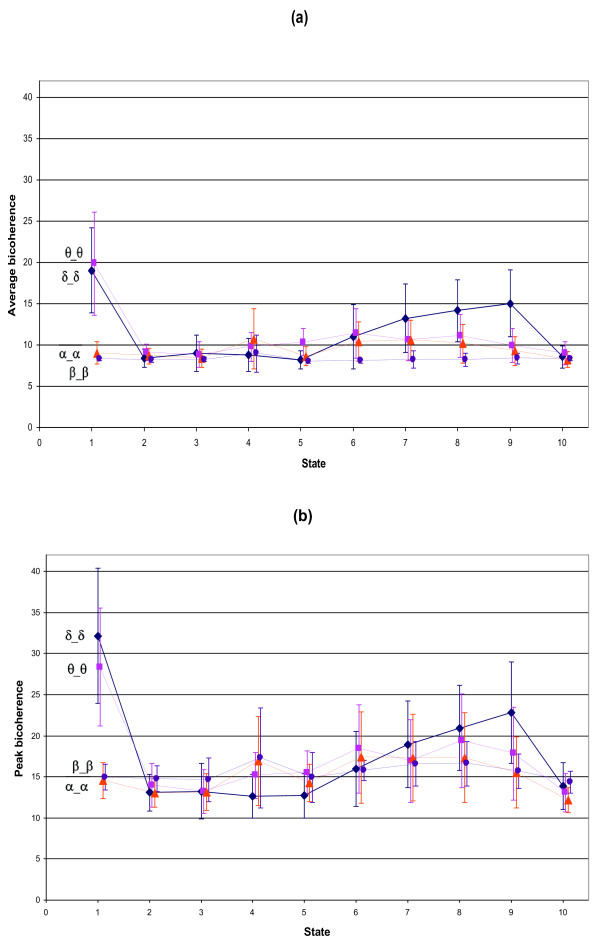
**Mean bicoherence at each EEG state**. Presenting the equal band bifrequency regions along the diagonal of the bicoherence plane, with one standard deviation for each observation. (a) Average bicoherence parameter and (b) peak bicoherence parameter.

Figure 3 shows the mean ± SD bicoherence value, of each state in the bifrequency regions along the diagonal (δ_δ, θ_θ, α_α and β_β), with both the average (a) and the peak (b) bicoherence parameters. There is a general trend of increasing bicoherence in lower bifrequency regions with deepening sedation. States 1 and 10 do not follow this trend, with state 1 resulting in high bicoherence values in the lower bifrequency regions and state 10 resulting in low bicoherence through all bifrequency regions.

Table 3 provides the z-value, p-value and intraclass correlation (ρ) of the univariate GLMs used to investigate whether the mean bicoherence values of conscious versus unconscious states were different, with eye movements and burst suppression (states 1 and 10) included (table 3a) and excluded (table 3b). Overall, the magnitudes of the z-values increased when states 1 and 10 were excluded from analysis. A negative z-value reflects a negative angle of slope for the linear regression.

**Table 3:** Parameters of the linear regression investigating differences in mean bicoherence values of consciousness versus unconsciousness.

(a) Bifrequency region	Averages			Peaks		
	z value	p-value	ρ	z value	p-value	ρ
δ_δ	-15.09	< 0.001	0.422	-15.05	< 0.001	0.388
δ_θ	-14.66	< 0.001	0.423	-17.25	< 0.001	0.417
δ_α	-6.64	< 0.001	0.369	-8.06	< 0.001	0.366
δ_β	-7.13	< 0.001	0.327	-6.67	< 0.001	0.200
θ_θ	-6.59	< 0.001	0.358	-10.75	< 0.001	0.404
θ_α	-6.09	< 0.001	0.437	-7.24	< 0.001	0.455
θ_β	-0.93	0.352	0.451	-3.05	0.002	0.319
α_α	-7.93	< 0.001	0.435	-9.05	< 0.001	0.444
α_β	-1.35	0.177	0.490	-6.79	< 0.001	0.385
β_β	2.85	0.004	0.692	0.75	0.453	0.632

(b) Bifrequency region	Averages			Peaks		
	z value	p-value	ρ	z value	p-value	ρ

δ_δ	-19.18	< 0.001	0.464	-20.17	< 0.001	0.432
δ_θ	-22.76	< 0.001	0.514	-22.88	< 0.001	0.473
δ_α	-12.87	< 0.001	0.449	-12.2	< 0.001	0.406
δ_β	-7.73	< 0.001	0.352	-7.06	< 0.001	0.203
θ_θ	-12.43	< 0.001	0.430	-14.54	< 0.001	0.447
θ_α	-8.4	< 0.001	0.471	-9.72	< 0.001	0.496
θ_β	-0.21	0.831	0.467	-2.71	0.007	0.341
α_α	-8.01	< 0.001	0.439	-9.29	< 0.001	0.456
α_β	-0.58	0.561	0.499	-6.51	< 0.001	0.390
β_β	3.72	< 0.001	0.723	1.34	0.181	0.656

(a) Including and (b) excluding eye movements and burst suppression. Linear regression performed using the univariate generalised linear mixed effects model where ρ is the intraclass correlation based on a random intercept model.

The ROC curves and prediction probabilities (P_k_) for distinguishing between the conscious and unconscious states were calculated when including or excluding periods of eye movements and burst suppression. Table 4 presents the values for the area under the ROC curve and P_k _(including 95% confidence interval) for the BIS index and the average and peak bicoherence parameters for each bifrequency region. This shows that the bicoherence values all have a smaller area under ROC curve and P_k _values than the BIS values. The δ_θ bifrequency region provides the greatest area under ROC curve and P_k _values for both the average and peak bicoherence parameters. The area under ROC curve and P_k _values both increase when the periods of eye movements and burst suppression are excluded, except for when using BIS values. Using the method described by Hanley and McNeil [34], the area under ROC curve values were compared for different parameters and are presented as follows, for the cases of including and excluding eye blinks and burst suppression. Between the δ_δ and δ_θ average bicoherence p-values were p = 0.736 and 0.311 respectively and for the peak bicoherence p-values were p = 0.485 and 0.206 respectively. Between average and peak δ_θ bicoherence, they were p = 0.234 and 0.070 respectively, and between average and peak δ_δ bicoherence they were p = 0.409 and 0.105 respectively. The area under the ROC curve values were significantly different between the BIS index and peak δ_θ bicoherence, with p-values of p < 0.001 and p = 0.003 respectively.

**Table 4:** Area under ROC curve and prediction probability (P_k_) estimates for determining consciousness versus unconsciousness.

Bifrequency region	Including eye blinks and burst suppression		Excluding eye blinks and burst suppression	
	Area under ROC	**P**_**k**_	Area under ROC	**P**_**k**_
Averages				
δ_δ	0.799 (0.765-0.833)	0.800 (0.766-0.834)	0.859 (0.836-0.882)	0.860 (0.838-0.883)
δ_θ	0.806 (0.767-0.844)	0.804 (0.765-0.842)	0.873 (0.849-0.896)	0.871 (0.848-0.894)
δ_α	0.679 (0.637-0.721)	0.679 (0.637-0.721)	0.726 (0.690-0.762)	0.726 (0.690-0.762)
δ_β	0.687 (0.646-0.728)	0.639 (0.601-0.678)	0.711 (0.672-0.751)	0.659 (0.621-0.697)
θ_θ	0.660 (0.619-0.701)	0.659 (0.618-0.700)	0.709 (0.674-0.745)	0.708 (0.673-0.744)
θ_α	0.634 (0.592-0.676)	0.630 (0.589-0.672)	0.668 (0.630-0.707)	0.664 (0.626-0.703)
θ_β	0.547 (0.504-0.589)	0.540 (0.502-0.579)	0.541 (0.499-0.584)	0.539 (0.500-0.577)
α_α	0.649 (0.610-0.688)	0.646 (0.607-0.684)	0.660 (0.620-0.700)	0.657 (0.617-0.696)
α_β	0.574 (0.535-0.613)	0.578 (0.541-0.614)	0.570 (0.530-0.309)	0.577 (0.540-0.614)
β_β	0.640 (0.598-0.681)	0.607 (0.571-0.643)	0.638 (0.595-0.681)	0.607 (0.569-0.645)
Peaks				
δ_δ	0.812 (0.777-0.848)	0.808 (0.773-0.844)	0.876 (0.854-0.898)	0.871 (0.849-0.893)
δ_θ	0.826 (0.789-0.863)	0.823 (0.786-0.860)	0.892 (0.870-0.914)	0.889 (0.867-0.911)
δ_α	0.688 (0.647-0.730)	0.686 (0.645-0.727)	0.739 (0.703-0.774)	0.735 (0.700-0.771)
δ_β	0.658 (0.613-0.703)	0.634 (0.592-0.677)	0.685 (0.641-0.729)	0.656 (0.613-0.698)
θ_θ	0.697 (0.659-0.735)	0.697 (0.659-0.734)	0.752 (0.721-0.783)	0.751 (0.721-0.781)
θ_α	0.658 (0.617-0.699)	0.650 (0.609-0.690)	0.697 (0.660-0.735)	0.687 (0.650-0.724)
θ_β	0.601 (0.558-0.644)	0.589 (0.549-0.630)	0.615 (0.571-0.659)	0.603 (0.562-0.644)
α_α	0.654 (0.617-0.691)	0.657 (0.620-0.693)	0.672 (0.633-0.710)	0.674 (0.635-0.712)
α_β	0.678 (0.639-0.717)	0.654 (0.616-0.692)	0.689 (0.649-0.729)	0.665 (0.626-0.704)
β_β	0.513 (0.442-0.532)	0.508 (0.467-0.550)	0.491 (0.444-0.537)	0.503 (0.460-0.546)
BIS index				
	0.941 (0.921-0.961)	0.935 (0.914-0.955)	0.935 (0.913-0.957)	0.928 (0.905-0.950)

Data are mean (95% confidence interval)

**Figure 4:**
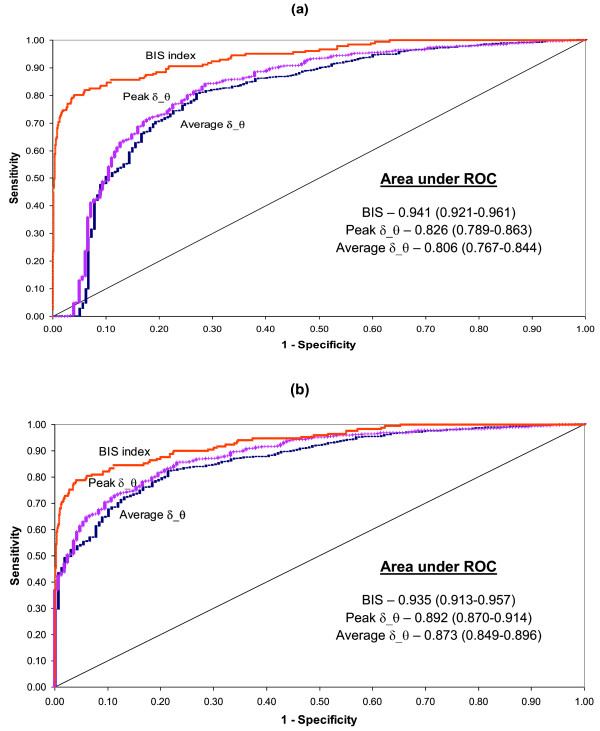
**ROC curve for consciousness versus unconsciousness**. Presenting ROC curves for BIS and the average and peak bicoherence values for the δ_θ bifrequency region, when (a) including and (b) excluding periods of eye blinks and burst suppression. δ_θ displayed the largest area under ROC values among all bifrequency regions. Area under ROC values are mean (95% confidence interval).

Figure 4 displays the ROC curves of BIS and the average and peak bicoherence estimates of the δ_θ bifrequency region for discriminating between consciousness and unconsciousness when (a) including and (b) excluding periods of eye movements and burst suppression. When eye movements and burst suppression are excluded, the peak and average curves are seen to be closer to the BIS curve than when the eye movements and burst suppression periods are included.

**Figure 5:**
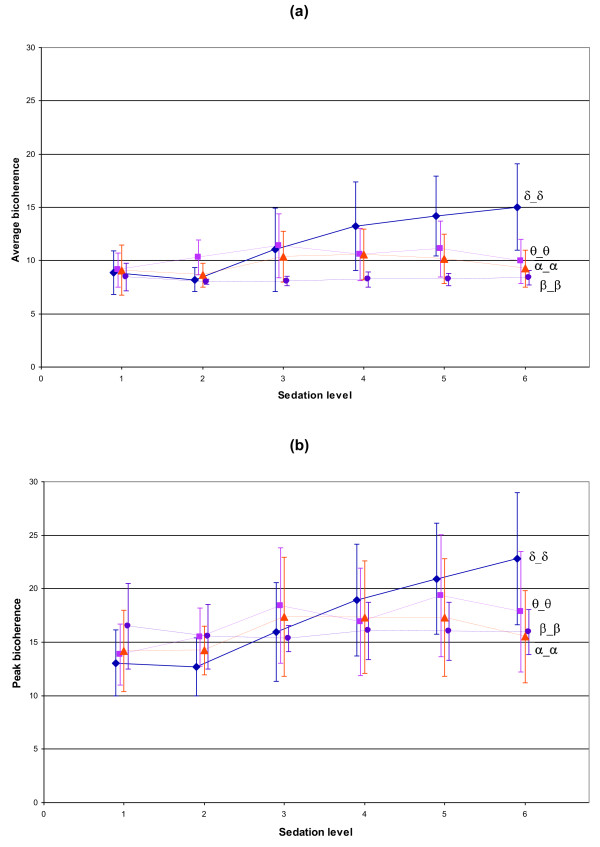
**Mean bicoherence at each level of anaesthesia**. Presenting the equal band bifrequency regions along the diagonal of the bicoherence plane, with one standard deviation for each observation. (a) Average bicoherence parameter and (b) peak bicoherence parameter.

Graduated levels of anaesthesia, as presented in table 5, were used to investigate the ability to monitor depth of anaesthesia using the chosen bicoherence analysis parameters. Figure 5 shows the mean ± SD bicoherence value at each level of anaesthesia in the bifrequency regions along the diagonal (δ_δ, θ_θ, α_α and β_β), with both the average (a) and the peak (b) bicoherence parameters. The bicoherence estimates are observed to increase with deepening levels of anaesthesia. Univariate GLMs have been performed on these graduated levels of anaesthesia for each bicoherence parameter. Table 6 presents the coefficient, 95% confidence interval, z-value, p-value, intraclass correlation (ρ) and R^2 ^of this model. The δ_θ bifrequency region provided the best fit of this model with the δ_δ bifrequency region providing the next best fit, however, the corresponding R^2 ^values are low in all instances (< 0.2).

**Table 5:** Summary of levels of anaesthesia, what EEG states they relate to and sample sizes.

Sedation level	Meaning	Including EEG states	Sample size	
			Number of observations	Number of subjects
1	Awake	2, 3 & 4	169	40
2	Drowsy/very light anaesthesia	5	11	5
3	Light anaesthesia	6	14	4
4	Moderate anaesthesia	7	444	31
5	Deep moderate anaesthesia	8	999	40
6	Deep anaesthesia	9	159	24

**Table 6:** Parameters of linear regression for different bicoherence estimates in association with graduated levels of anaesthesia.

(a) Bifrequency region	Coef (95% conf. interval)	z value	p-value	ρ	R^2^
δ_δ	1.217 (1.105 - 1.33)	21.27	< 0.001	0.462	0.152
δ_θ	0.985 (0.906 - 1.063)	24.61	< 0.001	0.509	0.168
δ_α	0.269 (0.227 - 0.311)	12.66	< 0.001	0.445	0.060
δ_β	0.066 (0.051 - 0.081)	8.67	< 0.001	0.356	0.036
θ_θ	0.403 (0.325 - 0.48)	10.21	< 0.001	0.426	0.044
θ_α	0.19 (0.145 - 0.236)	8.25	< 0.001	0.476	0.024
θ_β	0.007 (-0.01 - 0.024)	0.83	0.408	0.468	0.002
α_α	0.186 (0.115 - 0.258)	5.14	< 0.001	0.439	0.011
α_β	-0.002 (-0.024 - 0.019)	-0.21	0.831	0.499	0.002
β_β	-0.032 (-0.053 - -0.011)	-2.95	0.003	0.723	0.004

(b) Bifrequency region	Coef (95% conf. interval)	z value	p-value	ρ	R^2^

δ_δ	1.883 (1.728 - 2.038)	23.82	< 0.001	0.433	0.184
δ_θ	1.903 (1.758 - 2.048)	25.74	< 0.001	0.472	0.191
δ_α	0.786 (0.674 - 0.898)	13.74	< 0.001	0.396	0.072
δ_β	0.377 (0.304 - 0.449)	10.17	< 0.001	0.195	0.053
θ_θ	1.232 (1.069 - 1.394)	14.82	< 0.001	0.448	0.080
θ_α	0.582 (0.47 - 0.695)	10.18	< 0.001	0.502	0.036
θ_β	0.117 (0.05 - 0.184)	3.44	0.001	0.343	0.005
α_α	0.587 (0.432 - 0.742)	7.44	< 0.001	0.458	0.020
α_β	0.207 (0.128 - 0.287)	5.13	< 0.001	0.391	0.012
β_β	-0.059 (-0.133 - 0.015)	-1.56	0.119	0.656	0.008

(a) Average and (b) smoothed-peak bicoherence estimates. Linear regression performed using the univariate generalised linear mixed effects model where ρ is the intraclass correlation based on a random intercept model.

The P_k _values were found for the ability to determine between the graduated levels of anaesthesia using the average and peak bicoherence estimates and BIS values. The resulting P_k _(95% confidence interval) values are presented in table 7. The δ_θ bifrequency region provides the greatest P_k _value when using either the average or peak bicoherence (0.701 (0.681-0.721) and 0.709 (0.689-0.729) respectively). BIS provides a considerably greater P_k _value than these at 0.801 (0.786-0.816).

**Table 7:** P_k _values for the classification of graduated levels of anaesthesia.

Bifrequency region	**P**_**k**_	
	Averages	Peaks
δ_δ	0.658 (0.636 - 0.680)	0.688 (0.667 - 0.709)
δ_θ	0.701 (0.681 - 0.721)	0.709 (0.689 - 0.729)
δ_α	0.604 (0.583 - 0.625)	0.649 (0.628 - 0.670)
δ_β	0.565 (0.544 - 0.586)	0.656 (0.613 - 0.698)
θ_θ	0.557 (0.535 - 0.580)	0.620 (0.599 - 0.642)
θ_α	0.559 (0.538 - 0.581)	0.575 (0.554 - 0.597)
θ_β	0.537 (0.516 - 0.557)	0.548 (0.527 - 0.568)
α_α	0.505 (0.483 - 0.528)	0.527 (0.505 - 0.549)
α_β	0.504 (0.483 - 0.525)	0.528 (0.507 - 0.550)
β_β	0.518 (0.496 - 0.539)	0.500 (0.479 - 0.522)
		
P_k _for BIS index		
0.801 (0.786 - 0.816)		

Data are mean (95% confidence interval)

The differences between the bicoherence estimates during eye movement artifacts (state 1) and burst suppression (state 10) versus other physiological states, was examined using univariate GLMs. Table 8 presents the bicoherence parameters and bifrequency regions (with respective z and p-values) that yielded the largest differences when states 1 and 10 were compared to all other states. It was found that the highest |z| values (and therefore greatest distinguishability) for eye movements most often resided in the δ_θ bifrequency region, using the average bicoherence parameter. When comparing burst suppression to each of the other states (excluding eye movements), it was noted that the largest |z| values were found with comparison to the deeper anaesthetic levels (states 8 and 9).

**Table 8:** Estimates of difference between eye movements or burst suppression with respect to all other states.

State pair	z value	p-value	ρ	Bifrequency region	Parameter
1,2	-9.53	< 0.001	0.469	δ_θ	Peak
1,3	-21.98	< 0.001	0.075	δ_θ	Average
1,4	-13.09	< 0.001	0.564	δ_θ	Average
1,5	-7.07	< 0.001	0.895	δ_θ	Average
1,6	-4.84	< 0.001	0.384	δ_δ	Peak
1,7	-19.25	< 0.001	0.475	δ_θ	Average
1,8	-17.48	< 0.001	0.518	δ_α	Average
1,9	-10.04	< 0.001	0.333	θ_θ	Average
1,10	-15.82	< 0.001	0.891	δ_θ	Average
2,10	4.12	< 0.001	0.459	δ_β	Average
3,10	8.17	< 0.001	0.2	δ_β	Average
4,10	-3.81	< 0.001	0.145	θ_θ	Peak
5,10	3.9	< 0.001	< 0.001	β_β	Average
6,10	-3.85	< 0.001	0.455	θ_θ	Peak
7,10	-4.9	< 0.001	0.682	α_α	Peak
8,10	-12.12	< 0.001	0.654	δ_θ	Average
9,10	-6.79	< 0.001	0.546	δ_δ	Average

State 1 refers to eye movements and state 10 refers to burst suppression. This table shows the bicoherence parameter that contains the greatest discrimination for each pair of EEG states investigated, and the corresponding z value, p-value, ρ (intraclass correlation) and bifrequency region.

## Discussion

This study investigated changes in average and smoothed-peak bicoherence estimates during different states of anaesthetic depth as determined by visual inspection of the EEG. The δ_θ bifrequency region was the most sensitive to anaesthetic depth changes compared to other bifrequency regions.

Mean bicoherence values at each EEG state were inspected visually (Figure 3). It was noted that the biggest changes in the bicoherence values across all states, for both the average and peak parameters, occurred in the lower bifrequency regions. Viewing the δ_δ bifrequency region in particular, it could be seen that states 1 (eye movements) and 10 (burst suppression) did not appear to follow the same trend as the rest of the states. If states 1 and 10 were to be excluded, it could be seen that findings were consistent with that found by previous researchers [18], in that the bicoherence increased with deepening anaesthesia in the lower bifrequency range.

As discussed in the methods section, states 1 - 4 were collectively grouped as conscious and states 5 - 10 were grouped as unconscious. Although sustained alpha activity is usually a sign of the waking state in routine EEG, this pattern is normally predominant in the occipital region, spreading anteriorly with drowsiness, and has been noted to rarely be seen from the frontal electrodes used during depth of anaesthesia monitoring in awake patients [31]. As such, state 5 (alpha only) was included as an unconscious state during this analysis. It is of interest to note that, in this study, EEG patterns during the anaesthetised periods generally contained sustained alpha-like activity. This is in contrast to what would be expected during natural sleep, where alpha activity ceases and it is likely that the alpha-like activity observed is related to the spindle waves generated by the thalamocortical network [31,36].

Linear regression by means of univariate GLMs were used to investigate if the bicoherence parameters relating to conscious and unconscious levels were different, where eye movements and burst suppression were included (table 3a) and excluded (table 3b). The peak bicoherence generally provided a greater resolution (higher magnitude z values) than the average bicoherence estimates. Excluding periods of eye movements and burst suppression from the analysis increased the difference between bicoherence values at consciousness and unconsciousness, particularly in the lower bifrequency regions. This was expected as periods with eye movements contained higher bicoherence values than the rest of the awake states and vice-versa for periods with burst suppression. The greatest |z| values were generally associated with the δ_θ bifrequency region, except for when average bicoherence values were used with eye movements and burst suppression periods included in the analysis. In this instance, the δ_δ bifrequency area provided the greater |z| value. These values were always greater in the δ_δ and δ_θ bifrequency areas than anywhere else.

To test the performance of using bicoherence estimates as a diagnostic test for consciousness, the area under the ROC curve and prediction probabilities (P_k_) of the bicoherence estimates were compared to that of BIS. The δ_θ bifrequency region provided the greatest area under the ROC curve (although not significantly greater than δ_δ) for both the average and peak bicoherence parameters with 0.806 (0.767-0.844) and 0.826 (0.789-0.863) respectively when including eye movements and burst suppression, and 0.873 (0.849-0.896) and 0.892 (0.870-.914) respectively when excluding these patterns (table 4). The area under the ROC curve values for bicoherence were significantly smaller than those for the BIS values, which were 0.941 (0.921-0.961) when including and 0.935 (0.913-0.957) when excluding eye movements and burst suppression. The prediction probability had similar results. It was not unexpected that BIS index values would produce a greater area under the ROC curve value than the bicoherence parameters, as the BIS index had been developed with a combination of analytical methods in order to maximise the predictive capabilities of monitoring anaesthetic depth [37]. The drop in the area under ROC curves and P_k _values observed in the BIS data when excluding periods of eye movements and burst suppression was not unreasonable considering that periods such as burst suppression are incorporated when BIS index is calculated. The increase in the area under ROC and P_k _values observed when using either the average or peak bicoherence can be more fully explained by observing the ROC curves. The ROC curves for both the peak and average bicoherence of the δ_θ bifrequency region when including eye movements and burst suppression started with a horizontal segment, where zero sensitivity was associated with specificity below 100 (Figure 4a). Therefore a scenario was possible where there were false positives even with zero sensitivity. This was due to the fact that the highest bicoherence values seen in state 1 were related to consciousness and the next highest bicoherence values in state 9 related to unconsciousness (refer to the δ_δ bifrequency region in Figure 3, which had a similar configuration to the δ_θ bifrequency region). This effect was not observed once the periods of eye movements and burst suppression were excluded (Figure 4b), indicating that EEG involving artifacts or abnormal patterns adversely influence the ability to use bicoherence alone as a monitor for depth of anaesthesia. Therefore, considering the improvements in fitting the statistical models used, eye movements and burst suppression patterns were chosen to be excluded from further analysis.

Meaningful levels of anaesthesia were defined (table 5), as described in the results section, and linear regressions by means of univariate GLMs were performed on these levels to investigate the association of bicoherence values with graduated levels of anaesthesia. Looking at the |z| and R^2 ^values of the GLMs (table 6), it was again seen that the δ_δ and δ_θbifrequency regions provided the best fit. The |z| and R^2 ^values were higher in the δ_θ area than the δ_δ area. The |z| and R^2 ^values were also higher using the peak rather than the average parameter.

The ability to track graduated changes in levels of anaesthesia using the peak or average bicoherence parameters was tested using P_k _and compared to that of the BIS index (table 7). The P_k _value of BIS for distinguishing between graduated levels of anaesthesia is lower than that for distinguishing between consciousness and unconsciousness (0.801 (0.786-0.816) and 0.928 (0.905-0.950) respectively). This is to be expected, as there is an increased number of levels that need to be discriminated in the analysis of the graduated anaesthetic levels compared to consciousness versus unconsciousness and therefore there is a greater chance of overlap in the BIS values between levels. The P_k _values for the average and peak bicoherence values were largest in the δ_θ bifrequency region (0.701 (0.681-0.721) and 0.709 (0.689-0.729) respectively) and these values are significantly smaller than the BIS P_k _value. This indicated that the use of either the average or peak bicoherence value of any one bifrequency region on its own to monitor anaesthetic depth was inferior to BIS, even after artifacts and abnormal EEG were removed from analysis.

Throughout this analysis it has been observed that the greatest changes in bicoherence, associated with EEG patterns relating to anaesthetic depth, have occurred in the δ_δ and δ_θ bifrequency regions. As was expected, these results support work by Hayashi et al [24], who have analysed the EEG bicoherence in relation to drug concentration during general anaesthesia. As discussed in the introduction, their work has focused on changes along the diagonal of the bicoherence plane. They have shown the development of a peak in the low frequency region of the diagonal (between 2 and 6 Hz), which coincides with the changes seen in the current study in the δ_δ bifrequency region. They have also commented on the fact that the low frequency peak along the diagonal formed part of a larger broad peak that spread out into the δ and θ regions away from the diagonal. The current study has expanded on these results, indicating that the bicoherence peaks seen away from the diagonal (corresponding with the unequal band δ_θ bifrequency region) may provide greater statistical discrimination for tracking anaesthetic changes, than that of the diagonal (corresponding with the equal band δ_δ bifrequency region).

As states 1 and 10 were outliers in the trend of bicoherence values increasing with deepening anaesthesia, they have been largely excluded from the analysis. It was of interest, however, to investigate the bicoherence during these 2 states more thoroughly. State 1 refers to the condition where eye movements were present, producing repetitive, low frequency, sharp-like transients in the EEG. State 10 refers to the presence of burst suppression, and consists of periodic bursts of activity (generally recurring every 2 - 10 seconds), separated by intervals of isoelectric activity [38]. Table 8 shows the results of the univariate GLMs used to investigate how different the bicoherence values of state 1 and 10 are, in comparison with each other state.

Bicoherence from EEG with repetitive eye movement artifact (state 1) was found to have a higher value than all other EEG patterns investigated, particularly in the δ_θ bifrequency region and when average bicoherence was used (table 8). Bullock et al [32] indicated that transients of the same shape that are repeated in a signal, even if irregularly, would contribute towards increasing the bicoherence value. This accounts for the increased bicoherence seen at state 1 in the δ_δ and θ_θ bifrequency regions in Figure 3, and the higher bicoherence values found in the δ_θ bifrequency region, as indicated by the results of the GLMs in table 8. It may also indicate that bicoherence could be artificially raised by repetitive movement artifacts, if the movement artifacts were of a similar shape.

When comparing burst suppression (state 10) to each other state, it was noted that in general, the |z| values were lower than when comparing eye movements (state 1) to each other state. The largest |z| value was found with respect to one of the deeper anaesthetic levels (state 8, table 8) and there was no trend as to which bifrequency area produced the largest |z| value. It is known that during the burst activity of the burst suppression period, bicoherence is increased [39], however, it is assumed that during the suppressed activity of the burst suppression period, the bicoherence would be minimal, leading to overall low bicoherence values during burst suppression. These findings were consistent with biphasic behaviour reported in spectral parameters during burst suppression with respect to other patterns seen during deep anaesthesia [40].

During this study, anaesthetic practice was at the discretion of the anaesthetist. The majority of patients (83%) received a similar anaesthetic regimen of propofol bolus induction and sevoflurane maintenance, with the administration of midazolam, morphine, fentanyl or neuromuscular blockers as needed. Although there has been a report that BIS index will decrease in fully awake individuals with neuromuscular block [41], it is more likely that this is due to the (older version of the) BIS EMG/artifact processing than to changes in the EEG, which was later reported to be unaffected by the neuromuscular block administration [42,43]. Therefore, the latter 3 drug classes administered have no effect on the EEG in the doses used. There was, however, a variety of anaesthetic agents used as summarised in Table 1. It was considered that a protocol allowing for varying anaesthetic practice would be of benefit to the current analysis, as it provided a dataset associated with routine clinical anaesthesia and can thus maximise external validity of the findings. Although the results are not included in this paper, analysis was performed on the subset of patients that received the similar anaesthetic regimen, and it was found that the results were consistent with those found using the entire patient set. Further analysis is required on a larger data set incorporating a greater mix of anaesthetic regimens to more fully address the issue of varied anaesthetics.

Data was obtained for this study with minimal disruption to patient surgery, and as such the majority of the EEG recording for each subject was collected at the levels of anaesthesia best suited to surgery. Recording in this manner has resulted in uneven sample sizes for different EEG states, as seen in Table 2. As data was recorded in association with routine clinical anaesthesia, not all EEG states were observed in each subject, resulting in an uneven number of subjects for each EEG state.

It is unclear whether there is an abrupt transition from consciousness to unconsciousness (and vice-versa) when a critical threshold in EEG activity is reached, or if there is a continuous progression between these states. For this study, however, deepening anaesthesia levels have been treated as a continuous, perhaps even linear, process. Visual classification of the EEG is reported as a valuable tool in accessing depth of anaesthesia [30]. Therefore, after a review of the available literature relating to visual classification [6,28,30,31] and inspection of the raw EEG, the EEG states and graduated levels of anaesthesia have been selected, as described in Figure 1 and Tables 2 and 5, with the aim of simplified repeatability for EEG reviewers that may not possess specialist training.

Bullok at al [32] indicated that bicoherence fluctuates, even when similar patterns are present in the EEG visually, which would suggest that high variability should be expected when analysing EEG bicoherence. Hayashi et al [19] showed that to reduce the variability found in the bicoherence, it was more appropriate to use a 3 minute segment of EEG data with the same epoch length and overlap as used in this study. As data was collected for this study in a manner that would result in the least amount of disturbance to patient surgery, it is very impractical to expect to collect a minimum of 3 uninterrupted, artifact free minutes of EEG in each classified EEG state, in each patient. Therefore a 1 minute segment length was chosen, as this allowed a greater number of artifact free segments to be available for analysis, particularly for the awake and lighter sedation states. This may explain the large confidence intervals, and variability seen in the results.

## Conclusions

This study quantified associations between visually determined EEG patterns relating to deepening anaesthesia, and changes in bicoherence estimates for different frequency components and bicoherence estimation methods. The greatest changes occurred in the δ_δ and δ_θ bifrequency regions, and when burst suppression and large transients were excluded, the δ_θ peak bicoherence provided the greater area under the ROC curve and P_k _values for discrimination between conscious and unconscious states, and was significantly associated with graduated levels of anaesthesia. These results were supported by regression estimates from the generalised linear mixed effects statistical models. Increase in bicoherence was also established during episodes of eye movement artifacts. While identified associations extend earlier findings of bicoherence changes with increases in anaesthetic drug concentration, results indicate that ignoring the unequal band bifrequency regions of the bifrequency plane may diminish the predictive capabilities in the application of anaesthetic depth monitoring. The accuracy of bicoherence estimates alone in monitoring anaesthetic depth is inferior compared to BIS even after excluding burst suppression and artifact patterns.

## Abbreviations

EEG: Electroencephalogram; GLM: Generalised linear mixed effects model.

## Competing interests

The authors declare that they have no competing interests.

## Authors' contributions

All authors contributed towards the study protocol design, and critical revision of this manuscript. SP, ZMX and PM assisted in collection of data. SP, ZMX and EZ all contributed toward data analysis. SP drafted the manuscript. All authors have read and approved the final manuscript.

## Supplementary Material

Additional file 1 **a file explaining the steps of estimating bicoherence**. Click here for file

## References

[B1] Myles PS Symons JA Leslie K Anaesthetists' Attitudes Towards Awareness and Depth-of-Anaesthesia Monitoring Anaesthesia 2003 58 11 16 10.1046/j.1365-2044.2003.02955.x 12492663

[B2] Sackel DJ Anesthesia Awareness: An Analysis of its Incidence, the Risk Factors Involved, and Prevention J Clin Anesth 2006 18 483 485 10.1016/j.jclinane.2006.08.003 17126773

[B3] Monk TG Saini V Weldon C Sigl J Anesthetic Management and One-Year Mortality After Noncardiac Surgery Anesth Analg 2005 100 4 10 10.1213/01.ANE.0000147519.82841.5E 15616043

[B4] Freye E Levy JV Cerebral Monitoring in the Operating Room and the Intensive Care Unit: An Introductory for the Clinician and a Guide for the Novice Wanting to Open a Window to the Brain. Part I: The Electroencephalogram J Clin Monit Comput 2005 19 1 76 10.1007/s10877-005-0712-z 16167222

[B5] Levy WJ Intraoperative EEG Patterns: Implications for EEG Monitoring Anesthesiology 1984 60 430 434 10.1097/00000542-198405000-00007 6711855

[B6] Schultz B Grouven U Schultz A Automatic Classification Algorithms of the EEG Monitor Narcotrend for Routinely Recorded EEG Data from General Anaesthesia: a Validation Study Biomedizinische Technik 2002 47 9 13 11921636 10.1515/bmte.2002.47.1-2.9

[B7] Da Silva FL Niedermeyer E, Da Silva FL Dynamics of EEGs as Signals of Neuronal Populations: Models and Theoretical Considerations Electroencephalography - Basic Principles, Clinical Applications, and Related Fields 1999 4 Baltimore: Williams & Wilkins 76 92

[B8] Rampil IJ A Primer for EEG Signal Processing in Anesthesia Anesthesiology 1998 89 980 1002 10.1097/00000542-199810000-00023 9778016

[B9] Bruhn J Ropcke H Hoeft A Approximate Entropy as an Electroencephalographic Measure of Anesthetic Drug Effect during Desflurane Anesthesia Anesthesiology 2000 92 715 726 10.1097/00000542-200003000-00016 10719951

[B10] Viertio-Oja H Maja V Sarkela M Talja P Tenkanen N Tolvanen-Laakso H Paloheimo M Vakkuri A Yli-Hankala A Merilainen P Description of the Entropy Algorithm as Applied in the Datex-Ohmeda S/5 Entropy Module Acta Anaesthesiol Scand 2004 48 154 161 10.1111/j.0001-5172.2004.00322.x 14995936

[B11] Li X Cui S Voss LJ Using Permutation Entropy to Measure the Electroencephalographic Effects of Sevoflurane Anesthesiology 2008 109 448 456 10.1097/ALN.0b013e318182a91b 18719442

[B12] Olofsen E Sleigh JW Dahan A Permutation Entropy of the Electroencephalogram: a Measure of Anaesthetic Drug Effect Br J Anaesth 2008 101 810 821 10.1093/bja/aen290 18852113

[B13] Kearse L Saini V deBros F Chamoun N Bispectral Analysis of EEG May Predict Anesthetic Depth during Narcotic Induction Anesthesiology 1991 75 Suppl A175 10.1097/00000542-199109001-00175

[B14] Sebel PS Bowles S Saini V Chamoun N Accuracy of EEG in Predicting Movement at Incision during Isoflurane Anesthesia Anesthesiology 1991 75 Suppl A708 10.1097/00000542-199109001-00445

[B15] Vernon J Bowles S Sebel PS Chamoun N EEG Bispectrum Predicts Movement at Incision during Isoflurane or Propofol Anesthesia Anesthesiology 1992 77 Suppl A502 10.1097/00000542-199209001-00502

[B16] Lien CA Berman M Saini V Matteo RS Sharp GJ Chamoun N The Accuracy of the EEG in Predicting Hemodynamic Changes with Incision during Isoflurane Anesthesia Anesth Analg 1992 74 Suppl 187

[B17] Kearse LA Jr Manberg P DeBros F Chamoun N Sinai V Bispectral Analysis of the Electroencephalogram during Induction of Anesthesia may Predict Hemodynamic Responses to Laryngoscopy and Intubation Electroencephalogr Clin Neurophysiol 1994 90 194 200 10.1016/0013-4694(94)90091-4 7511501

[B18] Sigl JC Chamoun NG An Introduction to Bispectral Analysis for the Electroencephalogram J Clin Monit 1994 10 392 404 10.1007/BF01618421 7836975

[B19] Hagihira S Takashina M Mori T Mashimo T Yoshiya I Practical Issues in Bispectral Analysis of Electroencephalographic Signals Anesth Analg 2001 93 966 970 10.1097/00000539-200110000-00032 11574365

[B20] Hagihira S Takashina M Mori T Mashimo T Yoshiya I Changes of Electroencephalographic Bicoherence during Isoflurane Anesthesia Combined with Epidural Anesthesia Anesthesiology 2002 97 1409 1415 10.1097/00000542-200212000-00012 12459666

[B21] Hagihira S Takashina M Mori T Ueyama H Mashimo T Electroencephalographic Bicoherence Is Sensitive to Noxious Stimuli during Isoflurane or Sevoflurane Anesthesia Anesthesiology 2004 100 818 825 10.1097/00000542-200404000-00011 15087616

[B22] Morimoto Y Hagihira S Yamashita S Iida Y Matsumoto M Tsuruta S Sakabe T Changes in Electroencephalographic Bicoherence During Sevoflurane Anesthesia Combined with Intravenous Fentanyl Anesth Analg 2006 103 641 645 10.1213/01.ane.0000229699.99371.3c 16931674

[B23] Hayashi K Tsuda N Sawa T Hagihira S Ketamine Increases the Frequency of Electroencephalographic Bicoherence Peak on the {alpha} Spindle Area Induced with Propofol Br J Anaesth 2007 99 389 395 10.1093/bja/aem175 17621599

[B24] Hayashi K Sawa T Matsuura M Anesthesia Depth-dependent Features of Electroencephalographic Bicoherence Spectrum during Sevoflurane Anesthesia Anesthesiology 2008 108 841 850 10.1097/ALN.0b013e31816bbd9b 18431119

[B25] Tonner PH Balanced Anaesthesia Today Best Pract Res Clin Anaesthesiol 2005 19 475 484 10.1016/j.bpa.2005.02.005 16013695

[B26] Rao GSU Ali Z Ramamoorthy M Patil J Equi-MAC Concentrations of Halothane and Isoflurane do not Produce Similar Bispectral Index Values J Neurosurg Anesthesiol 2007 19 93 96 10.1097/ANA.0b013e31803062f9 17413994

[B27] Traast HS Kalkman CJ Electroencephalographic Characteristics of Emergence from Propofol/Sufentanil Total Intravenous Anesthesia Anesth Analg 1995 81 366 371 10.1097/00000539-199508000-00027 7618729

[B28] Kugler J Elektroenzephalographie in Klinik und Praxis 1981 Stuttgart, New York: Thieme

[B29] Johansen JW Update on Bispectral Index Monitoring Best Pract Res Clin Anaesthesiol 2005 20 81 99 10.1016/j.bpa.2005.08.004 16634416

[B30] Schultz A Grouven U Zander I Beger FA Siedenberg M Schultz B Age-Related Effects in the EEG During Propofol Anaesthesia Acta Anaesthesiol Scand 2004 48 27 34 10.1111/j.1399-6576.2004.00258.x 14674970

[B31] Bennett C Voss LJ Barnard JPM Sleigh JW Practical Use of the Raw Electroencephalogram Waveform during General Anesthesia: The Art and Science Anesth Analg 2009 109 539 550 10.1213/ane.0b013e3181a9fc38 19608830

[B32] Bullock TH Achimowicz JZ Duckrow RB Spencer SS Iragui-Madoz VJ Bicoherence of Intracranial EEG in Sleep, Wakefulness and Seizures Electroencephalogr Clin Neurophysiol 1997 103 661 678 10.1016/S0013-4694(97)00087-4 9546494

[B33] Smith WD Dutton RC Smith TN Measuring the Performance of Anesthetic Depth Indicators Anesthesiology 1996 84 38 51 10.1097/00000542-199601000-00005 8572353

[B34] Hanley JA McNeil BJ A method of comparing the areas under receiver operating characteristic curves derived from the same cases Radiology 1983 148 839 843 6878708 10.1148/radiology.148.3.6878708

[B35] Rabe-Hesketh S Skrondal A Pickles A Reliable Estimation of Generalized Linear Mixed Models using Adaptive Quadrature Stata Journal 2002 2 1 21

[B36] Kissin I Depth of Anesthesia and Bispectral Index Monitoring Anesth Analg 2000 90 1114 1117 10.1097/00000539-200005000-00021 10781463

[B37] Johansen JW Sebel PS Development and Clinical Application of Electroencephalographic Bispectrum Monitoring Anesthesiology 2000 93 1336 1344 10.1097/00000542-200011000-00029 11046224

[B38] Tatum WO Percy RC, Johnson R Patterns of Special Significance Handbook of EEG Interpretation 2007 USA: Demos Medical Publishing

[B39] Witte H Schack B Helbig M Putsche P Schelenz C Schmidt K Specht M Quantification of Transient Quadratic Phase Couplings within EEG Burst Patterns in Sedated Patients during Electroencephalic Burst-suppression Period Journal of Physiology Paris 2000 94 427 434 10.1016/S0928-4257(00)01086-X 11165910

[B40] Bruhn J Ropcke H Rehberg B Bouillon T Hoeft A Electroencephalogram Approximate Entropy Correctly Classifies the Occurrence of Burst Suppression Pattern as Increasing Anesthetic Drug Effect Anesthesiology 2000 93 981 985 10.1097/00000542-200010000-00018 11020750

[B41] Messner M Beese U Romstock J Dinkel M Tschaikowsky K The Bispectral Index Declines During Neuromuscular Block in Fully Awake Persons Anesth Analg 2003 97 488 491 10.1213/01.ANE.0000072741.78244.C0 12873942

[B42] Messner M Beese U Romstock J Dinkel M Tschaikowsky K Response to: A Low Voltage EEG Signal May Give Low Bispectral Index Values Anesthesia & Analgesia 2004 98 873 874

[B43] Messner M Beese U Romstock J Dinkel M Tschaikowsky K Response to: Bispectral Index Decline Caused by Neuromuscular Blockade Anesthesia & Analgesia 2004 98 871 872

