# Supplementary material for: Peak and averaged bicoherence for different EEG patterns during general anaesthesia
Source: Biomed Eng Online. 2010 Nov 20;9:76. doi: 10.1186/1475-925X-9-76 (PMC2998515; doi:10.1186/1475-925X-9-76)
Supplement: Additional file 1 — a file explaining the steps of estimating bicoherence. [file 1475-925X-9-76-S1.PDF]

Appendix

Calculating the Bicoherence

To find the bicoherence, first the bispectrum must be found. The bispectrum (*B*) depends on both the phase and amplitude relationships between two primary frequencies (f_1 & f_2) and their modulation component frequency ($f_1 + f_2$), as described in equation (1), where *X(f)* is the Fourier transform of the spontaneous EEG (x) and *X** is the complex conjugate of the Fourier transform. This group of frequencies is known as a triplet.

$$B(f_1, f_2) = X(f_1)X(f_2)X^*(f_1 + f_2) \tag{1}$$

The amplitude relationships are expressed in the real triple product (*RTP*) as described in equation (2), where *P* is the power of f ($|X(f)|^2$).

$$RTP(f_1, f_2) = P(f_1)P(f_2)P(f_1 + f_2) \tag{2}$$

The amplitude independent bicoherence (*BIC*) is calculated as the bispectrum normalized with respect to the *RTP* as described in equation (3). This results in a value between 0 and 100, indicating the percentage of phase correlation between the two frequencies, f_1 and f_2.

$$BIC(f_1, f_2) = 100\frac{B(f_1, f_2)}{\sqrt{RTP(f_1, f_2)}} \tag{3}$$

Due to the stochastic nature of the EEG, when calculating the bicoherence of a segment of EEG, it is important to average successive smaller epochs across that segment. As such, to calculate the bicoherence of a segment of EEG, the EEG needs

to be split into small epochs, which may be overlapped to increase the number of epochs available in a segment. The bicoherence is calculated for all epochs and then averaged, providing the average bicoherence for the original segment of EEG.

For further information regarding calculating bicoherence for depth of anaesthesia monitoring, please refer to either Rampil [1], or Sigl and Chamoun [2].

References:

1. Rampil IJ: **A Primer for EEG Signal Processing in Anesthesia.** *Anesthesiology* 1998, **89:**980-1002.

2. Sigl JC, Chamoun NG: **An Introduction to Bispectral Analysis for the Electroencephalogram.** *J Clin Monit* 1994, **10:**392-404.
